# Arbutin as a potential nephroprotective agent: Dose-related effects in renal ischemia–reperfusion injury

**DOI:** 10.17305/bb.2025.13056

**Published:** 2025-09-18

**Authors:** Ferhat Sirinyildiz, Izel Kavak, Nesibe Kahraman Cetin, Adem Keskin

**Affiliations:** 1 Department of Physiology, Faculty of Medicine, Aydin Adnan Menderes University, Aydin, Türkiye; 2 Department of Physiology, Institute of Health Sciences, Aydın Adnan Menderes University, Aydin, Türkiye; 3 Department of Medical Pathology, Faculty of Medicine, Aydin Adnan Menderes University, Aydin, Türkiye; 4 Department of Medical Biochemistry, Faculty of Medicine, Aydin Adnan Menderes University, Aydin, Türkiye

**Keywords:** Ischemia–reperfusion injury, arbutin, malondialdehyde, myeloperoxidase, glutathione peroxidase, glomerular necrosis, Bowman’s capsule dilation, interstitial hemorrhage

## Abstract

Ischemia–reperfusion injury (IRI) presents a complex pathophysiology characterized by oxidative stress and inflammation. Arbutin, widely recognized for its use in skin whitening, also exhibits antioxidant, anti-inflammatory, and anticancer properties. This study aimed to assess the potential protective effects of arbutin at two different doses against IRI in the kidneys. Twenty-four male Wistar albino rats were randomly assigned to four equal groups: Control, IRI, 250 mg/kg arbutin + IRI (AR250+IRI), and 1000 mg/kg arbutin + IRI (AR1000+IRI). Arbutin was administered orally via gavage for 14 days to ensure sub-acute application. Following left kidney nephrectomy, ischemia was induced in the right kidney using a non-traumatic clamp for 45 min, succeeded by 60 min of reperfusion. Blood and tissue samples were subsequently collected for analysis. In the IRI group, levels of malondialdehyde, myeloperoxidase, interleukin-1 beta, and creatinine were significantly elevated; these levels decreased in the groups receiving arbutin supplementation. Notably, ischemia-modified albumin, urea, superoxide dismutase (inhibition ratio), and tumor necrosis factor alpha levels were reduced in the AR1000+IRI group. Additionally, decreased levels of catalase and glutathione peroxidase were observed in the AR1000+IRI group. Histopathological examination revealed flattening, necrosis, degeneration, dilation, glomerular necrosis, sclerosis, Bowman capsule dilation, and interstitial hemorrhage in the IRI group. The AR250+IRI group exhibited mild cortical-medullary congestion and a slight increase in glomerular size. Conversely, the AR1000+IRI group displayed a histological appearance resembling that of the control group. In conclusion, arbutin demonstrates potential protective effects against IRI. Its use may be recommended prophylactically for individuals at risk of developing IRI.

## Introduction

Ischemia–reperfusion injury (IRI) is a significant health disorder that contributes to acute kidney injury (AKI), leading to rapid renal failure and increased mortality rates. IRI is frequently observed in contexts such as kidney transplantation, trauma, shock, and cardiovascular and urologic surgeries, yet there is currently no effective therapeutic intervention available [1]. The sudden and transient obstruction of renal blood flow associated with IRI results in high morbidity and mortality, highlighting the urgent need for effective treatment strategies [2]. Recent research has increasingly focused on identifying potential treatment or prevention strategies, given the complex pathophysiological processes underlying IRI and the absence of an established pharmacological remedy [3–7].

AKI encompasses a range of molecular and pathophysiological processes, including inflammation, oxidative stress, fibrosis, apoptosis, and altered gene expression that activate various signaling pathways. The cascade of inflammatory responses—including complement activation and the initiation of innate immunity—plays a crucial role in IRI. Additionally, IRI induces necrosis, apoptosis, oxidative damage to DNA and proteins, and peroxidation of membrane lipids [8]. Notably, there is an upregulation of proteins associated with specific pathways directly linked to ferroptosis and oxidative stress in the context of IRI. Ferroptosis and oxidative stress contribute to renal fibrosis, which may subsequently lead to chronic kidney disease [9]. Consequently, various therapeutic approaches have focused on mitigating oxidative stress, inflammation, and fibrosis [10].

Arbutin, a hydroquinone glycoside found in approximately 50 plant families, is one of the most widely utilized natural skin-lightening agents. Beyond its cosmetic applications, arbutin possesses several therapeutically relevant biological properties, including antioxidant, anti-inflammatory, antimicrobial, and anticancer activities [11]. It has been shown to reduce oxidative stress-induced cytotoxicity, which can result in significant tissue damage. Cells treated with arbutin exhibit a dose-dependent decrease in the production of reactive nitrogen and oxygen species [12]. Furthermore, arbutin demonstrates robust antioxidant and anti-inflammatory effects, with arbutin-containing microspheres inhibiting Nuclear Factor Kappa B (NF-κB) signaling and activating the nuclear factor erythroid 2-related factor 2 (Nrf2) pathway to exert antioxidative effects [13].

IRI, the predominant cause of AKI, is characterized by a complex pathophysiology driven by inflammation and oxidative stress, which complicates treatment efforts. While arbutin is recognized for its antioxidant and anti-inflammatory properties, its therapeutic and preventive effects on a broader spectrum of health disorders, particularly IRI, remain underexplored. This study aims to investigate the protective role of arbutin against IRI, leveraging its well-documented anti-inflammatory and antioxidant properties in the context of a condition characterized by oxidative stress and inflammation.

## Materials and methods

### Animals

The ethics committee approval necessary for this research was obtained from the Aydın Adnan Menderes University (ADU) Animal Experiments Local Ethics Committee (ADUHADYEK). This committee oversees the ethical conduct of experimental studies, ensuring compliance with established ethical guidelines. The experimental study involved 24 male Wistar albino rats, each weighing between 250–400 g, sourced from the ADU Faculty of Medicine Experimental Animal Production Center. The rats were randomly assigned to four groups using a simple random number table: a control group, an IRI group, a 250 mg/kg arbutin + IRI group (AR250+IRI), and a 1000 mg/kg arbutin + IRI group (AR1000+IRI), with an equal number of rats in each group.

### IRI formation and application of arbutin supplementation

In the control group, IRI was not induced in the right kidney. In contrast, IRI was induced in the other three groups by clamping the right kidney. Arbutin (≥98% purity, Sigma-Aldrich, St. Louis, MO, USA; product number Y0000806, European Pharmacopoeia Reference Standard) was freshly dissolved in distilled water immediately prior to administration. Prior to IRI, rats in the AR250+IRI group received an oral gavage of 250 mg/kg arbutin for 14 days (sub-acute application), while those in the AR1000+IRI group received 1000 mg/kg arbutin. The gavage volume was standardized to 1 mL per 100 g of body weight to ensure precise dosing across all animals.

**Figure 1.**
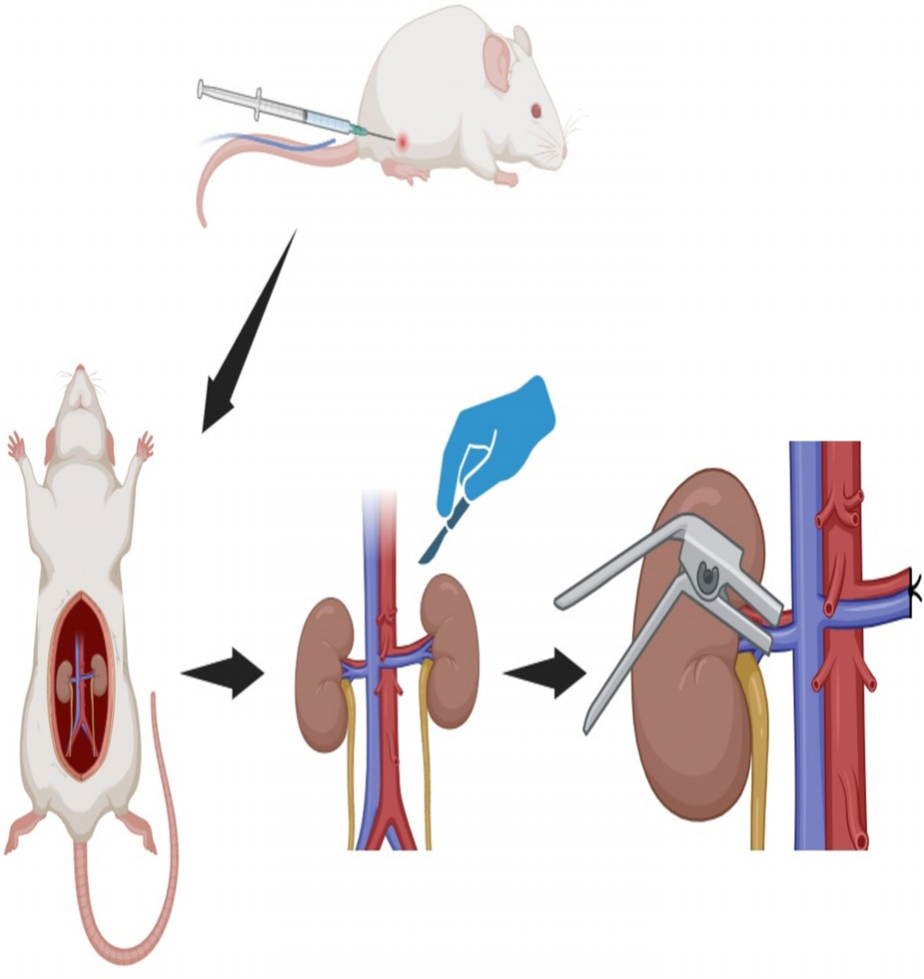
**Schematic view of the unilateral renal ischemia–reperfusion (IRI) model in rats.** After left kidney nephrectomy, ischemia was induced in the right kidney with a non-traumatic clamp for 45 min, followed by 60 min of reperfusion.

For the experimental renal IRI model, rats were anesthetized using intraperitoneal injections of ketamine (90 mg/kg; 60 mg/mL) and xylazine (10 mg/kg; 20 mg/mL). Following anesthesia, the abdominal cavity was opened, the left kidney was removed and ligated to prevent hemorrhage, leaving only the right kidney intact. A non-traumatic clamp was then applied to the right renal artery, resulting in a 45-min ischemia period followed by a 60-min reperfusion period (Figure 1). The 45-min ischemia duration was selected because renal histological damage and functional impairment are reliably observed after this period. For instance, a study by Kapisiz et al. [14] indicated that damage began within four hours and peaked 24 h after a 45-min ischemic event. Many studies in the literature on renal IRI favor protocols involving 45 min of ischemia followed by 60 min of reperfusion [15–18]. At the conclusion of the reperfusion phase, intracardiac blood samples were collected, and kidney tissue samples were preserved at –80 ^∘^C for subsequent biochemical analyses.

### Biochemical analyses

During biochemical analyses, the laboratory team was blinded to the sample group assignments. Blood samples were centrifuged at 3000 rpm for 10 min, and analyses of creatinine and urea were performed on the serum obtained from centrifugation. Tissue samples, thawed at room temperature, were homogenized in a phosphate buffer (50 mM, pH 7.0) at a ratio of 1:10 (w/v). The resulting tissue homogenates were then centrifuged at 4 ^∘^C for 15 min. Protein concentrations in the supernatants were quantified using the Bradford method, and all ELISA results were normalized to protein content. ELISA assays were conducted in duplicate following the manufacturers’ protocols, and mean values were employed for statistical analyses.

Supernatants obtained post-centrifugation were analyzed for malondialdehyde (K739-100, Biovision, USA) [19], superoxide dismutase (SOD) inhibition rate (K335-100, Biovision, USA) [20], myeloperoxidase (K744-100, Biovision, USA) [21], glutathione peroxidase (GPx) (K762-100, Biovision, USA) [22], tumor necrosis factor alpha (TNF-α) (K1052-100, Biovision, USA) [23], interleukin-1 beta (IL-1β) (K4796-100, Biovision, USA) [24], and ischemia-modified albumin (IMA) (ER1108, FineTest, China) [25]. The protocols of the respective assay kits were strictly followed, and readings were conducted using the Enzyme-Linked Immunosorbent Assay device.

**Table 1:** Biochemical parameters of the study groups

Parameters*	**Control**	**IRI**	**AR250+IRI**	**AR1000+IRI**
MDA (nmol/g)	286.83±40.24^a^	428.00±55.71^b^	335.50±54.39^a^	294.17±56.06^a^
MPO (U/g)	375.83±46.63^a^	631.33±62.29^b^	474.00±83.34^c^	422.33±33.11^a,c^
IMA (ng/g)	14.83±4.79^a^	21.83±3.87^b^	19.33±3.14^a,^^b^	15.83±2.14^a^
SOD inhibition (%)	14.50±5.09^a^	32.17±6.31^b^	22.83±7.36^a,b^	19.67±4.23^a^
CAT (U/g)	445.50±76.61^a^	220.50±68.04^b^	330.67±78.65^c^	375.83±43.53^a,c^
GPx (U/g)	799.17±92.38^a^	523.00±124.72^b^	561.17±92.88^b,c^	708.83±103.68^a,c^
TNF-α (pg/g)	559.50±78.53^a^	766.17±131.67^b^	654.67±61.86^a,b^	586.00±93.17^a^
IL-1β (pg/g)	438.33±126.18^a^	733.83±96.07^b^	539.50±53.07^a^	561.83±123.37^a^
Creatinine (mg/dL)	0.46±0.09^a^	0.95±0.15^b^	0.76±0.13^c^	0.68±0.09^c^
Urea (mg/dL)	13.67±3.76^a^	34.33±6.25^b^	27.83±3.06^b,c^	25.00±2.68^c^

Different superscript letters (^a,^ ^b,^ ^c^) in the same row indicate statistically significant differences between groups (*P* < 0.05). Intergroup differences were analyzed by one-way ANOVA with Tukey’s post-hoc test. *Creatinine and urea results were obtained from blood serum, while other parameters were obtained from tissue homogenate and normalized according to tissue quantity. Abbreviations: IRI: Ischemia-reperfusion injury; AR250: 250 mg/kg Arbutin; AR1000: 1000 mg/kg Arbutin; MDA: Malondialdehyde; MPO: Myeloperoxidase; IMA: Ischemia modified albumin; SOD: Superoxide dismutase (Inhibition rate); CAT: Catalase; GPx: Glutathione peroxidase; TNF-α: Tumor necrosis factor alpha; IL-1β: Interleukin-1 beta.

### Histopathological analyses

For histopathological analyses, tissue samples were fixed in 10% neutral formaldehyde at 24 ^∘^C. The tissues were subsequently embedded in paraffin blocks, which were then sectioned at a thickness of 5 microns using a microtome. Hematoxylin and eosin (H&E) staining was performed using a routine staining method. The sections were examined under a standard light microscope.

For H&E staining, the samples were immersed in xylene for a total of 9 min, with an initial immersion in xylene pools for 5 min followed by an additional 4 min. The samples were then washed in 100% and 80% alcohol solutions for 2 min each, followed by rinsing with distilled water. To prepare for microscopic examination, all samples were immersed in hematoxylin stain for 3 min, washed, and subsequently immersed in eosin stain for 1 min.

### Ethical statement

The ethics committee approval for this study was obtained from ADUHADYEK (Decision Number: 64583101/2023/05). The research was conducted and reported in compliance with the ARRIVE guidelines to ensure transparent and comprehensive reporting of animal experiments [26].

### Statistical analysis

IBM SPSS Statistics version 22.0 (IBM Corp., Armonk, NY, USA) was utilized for statistical analyses. Biochemical variables were assessed for normal distribution using the Shapiro–Wilk test and for homogeneity of variances using Levene’s test. Data are presented as mean ± standard deviation. Intergroup differences in the variables were analyzed through one-way ANOVA, followed by Tukey’s post-hoc test for multiple comparisons. Furthermore, Pearson correlation analysis was conducted. All statistical procedures adhered to established biostatistical standards [27].

## Results

### Effect of IRI on biochemical parameters

**Figure 2.**
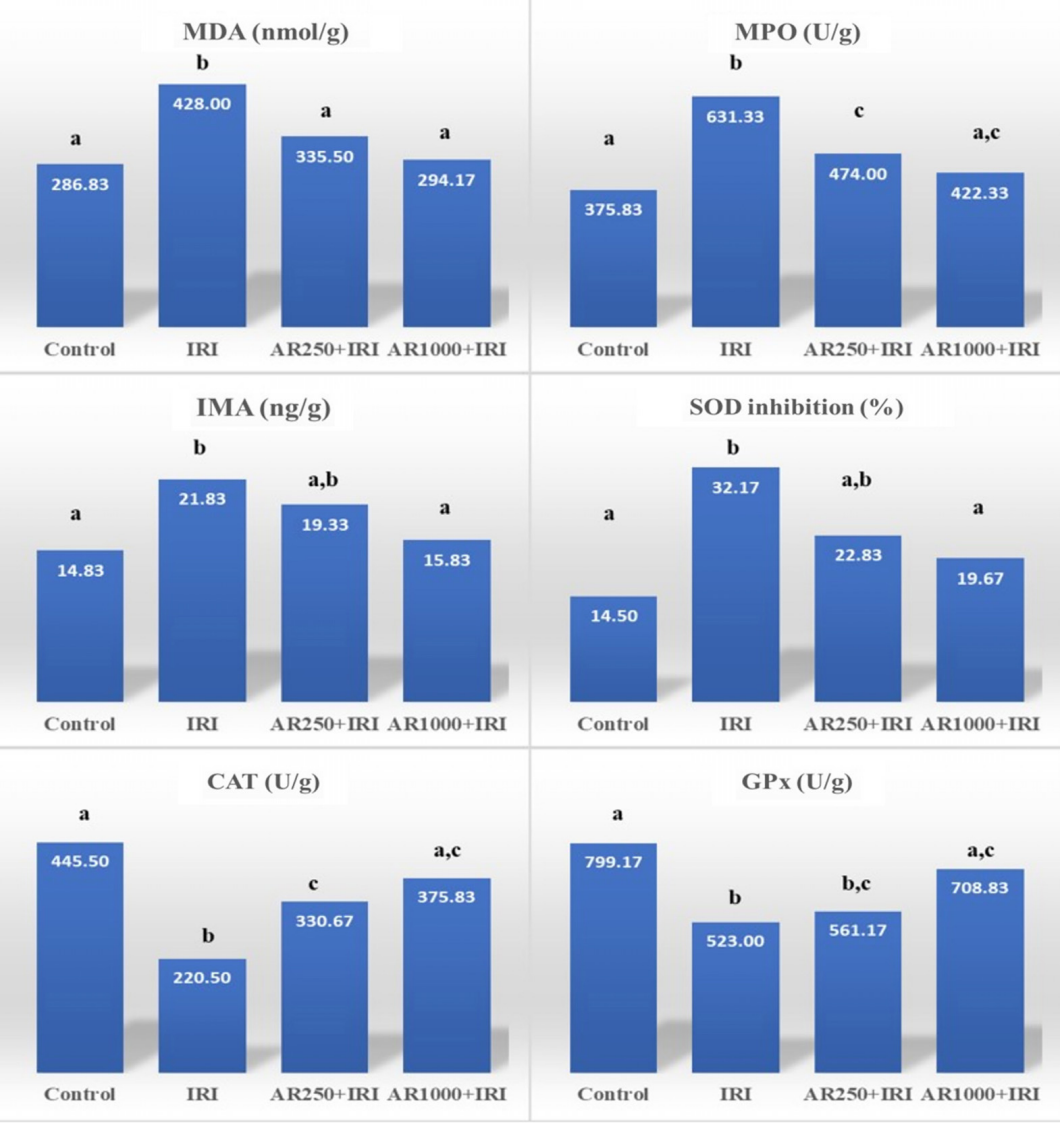
**Bar graphs of biochemical results of groups-1.** Different superscript letters (^a^, ^b^, ^c^) in the same row indicate statistically significant differences between groups (*P* < 0.05). Intergroup differences were analyzed by one-way ANOVA with Tukey’s post-hoc test. *Creatinine and urea results were obtained from blood serum, while other parameters were obtained from tissue homogenate and normalized according to tissue quantity. Abbreviations: MDA: Malondialdehyde; MPO: Myeloperoxidase; IMA: Ischemia modified albumin; SOD: Superoxide dismutase (inhibition rate); CAT: Catalase; GPx: Glutathione peroxidase.

**Figure 3.**
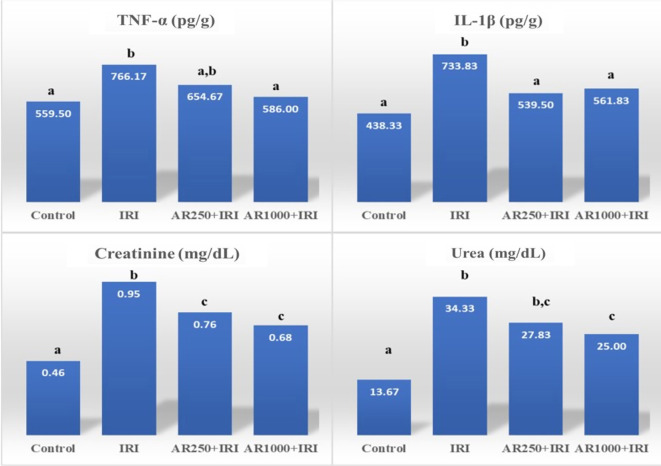
**Bar graphs of biochemical results of groups-2.** Different superscript letters (^a^, ^b^, ^c^) in the same row indicate statistically significant differences between groups (*P* < 0.05). Intergroup differences were analyzed by one-way ANOVA with Tukey’s post-hoc test. *Creatinine and urea results were obtained from blood serum, while other parameters were obtained from tissue homogenate and normalized according to tissue quantity. Abbreviations: TNF-α: Tumor necrosis factor alpha; IL-1β: Interleukin-1 beta.

Malondialdehyde, myeloperoxidase, IMA, tumor necrosis factor-alpha (TNF-α), IL-1β, creatinine, urea, and SOD inhibition rates exhibited the highest averages in the IRI group, while the control group demonstrated the lowest averages. Specifically, the IRI group had significantly elevated levels of malondialdehyde, myeloperoxidase, IMA, TNF-α, IL-1β, creatinine, urea, and SOD inhibition rates compared to the control group (*P* ═ 0.001, *P* < 0.001, *P* ═ 0.015, *P* ═ 0.006, *P* < 0.001, *P* < 0.001, *P* < 0.001, respectively) (see Table 1, Figures 2 and 3). Conversely, the lowest values for GPx and catalase enzyme activity were recorded in the IRI group, with the highest mean values observed in the control group. The enzyme activity levels of catalase and GPx were significantly lower in the IRI group compared to the control group (*P* ═ 0.001, *P* < 0.001, respectively) (refer to Table 1 and Figure 2).

### Effect of different doses of arbutin supplementation on IRI

Malondialdehyde, myeloperoxidase, IL-1β, and creatinine levels were significantly lower in the AR250+IRI group compared to the IRI group (*P* ═ 0.028, *P* ═ 0.001, *P* ═ 0.020, *P* ═ 0.042, respectively) (Table 1, Figures 2 and 3). Additionally, catalase enzyme activity was greater in the AR250+IRI group than in the IRI group (*P* ═ 0.049) (Table 1 and Figure 2).

In the AR1000+IRI group, malondialdehyde, myeloperoxidase, IMA, TNF-α, IL-1β, creatinine, urea, and SOD inhibition rates were significantly lower compared to the IRI group (*P* ═ 0.001, *P* < 0.001, *P* ═ 0.043, *P* ═ 0.021, *P* ═ 0.043, *P* ═ 0.004, *P* ═ 0.005, *P* ═ 0.007, respectively) (Table 1, Figures 2 and 3). Furthermore, catalase and GPx enzyme activity levels were higher in the AR1000+IRI group than in the IRI group (*P* ═ 0.004, *P* ═ 0.027, respectively) (Table 1 and Figure 2).

### Comparison of AR250+IRI and AR1000+IRI groups with each other and with the control group

Creatinine and urea levels were significantly elevated in the AR1000+IRI group relative to the control group (*P* ═ 0.025 and *P* < 0.001, respectively) (Table 1 and Figure 3).

Similarly, myeloperoxidase, creatinine, and urea levels were higher in the AR250+IRI group compared to the control group (*P* ═ 0.044, *P* ═ 0.002, and *P* < 0.001, respectively) (Table 1, Figures 2 and 3). Conversely, catalase and GPx enzyme activity levels were significantly lower in the AR250+IRI group compared to the control group (*P* ═ 0.004 and *P* ═ 0.039, respectively) (Table 1 and Figure 2).

### Correlation of biochemical findings with group scoring

The groups were categorized as follows: 1 = IRI group, 2 = Low-dose Arbutin + IRI (AR250 + IRI) group, 3 = High-dose Arbutin + IRI (AR1000 + IRI) group, and 4 = Healthy control group. Correlation analysis was conducted to examine the relationships between biochemical findings and group scores. The results of this analysis are presented in Table 2. A negative correlation was identified between group scores and levels of malondialdehyde, myeloperoxidase, IMA, TNF-α, IL-1β, and the inhibition rate of SOD, as well as creatinine and urea levels. Conversely, a positive correlation was observed between levels of catalase and GPx (Table 2).

**Table 2:** Correlation analysis results of group scores and biochemical findings

**Parameters**	Group score*	
	**Correlation coefficient**	***P***
Malondialdehyde	−0.706	<0.001
Myeloperoxidase	−0.828	<0.001
Ischemia modified albumin	−0.633	0.001
Superoxide dismutase (inhibition rate)	−0.750	<0.001
Catalase	0.745	<0.001
Glutathione peroxidase	0.784	<0.001
Tumor necrosis factor alpha	−0.653	0.001
Interleukin-1 beta	−0.678	<0.001
Creatinine	−0.837	<0.001
Urea	−0.866	<0.001

*The groups were scored as follows: 1 = IRI group, 2 = Low-dose Arbutin+IRI (AR250+IRI) group, 3 = High-dose Arbutin+IRI (AR1000+IRI) group, and 4 = Healthy control group. Abbreviation: IRI: Ischemia–reperfusion injury.

### Histopathological findings

**Figure 4.**
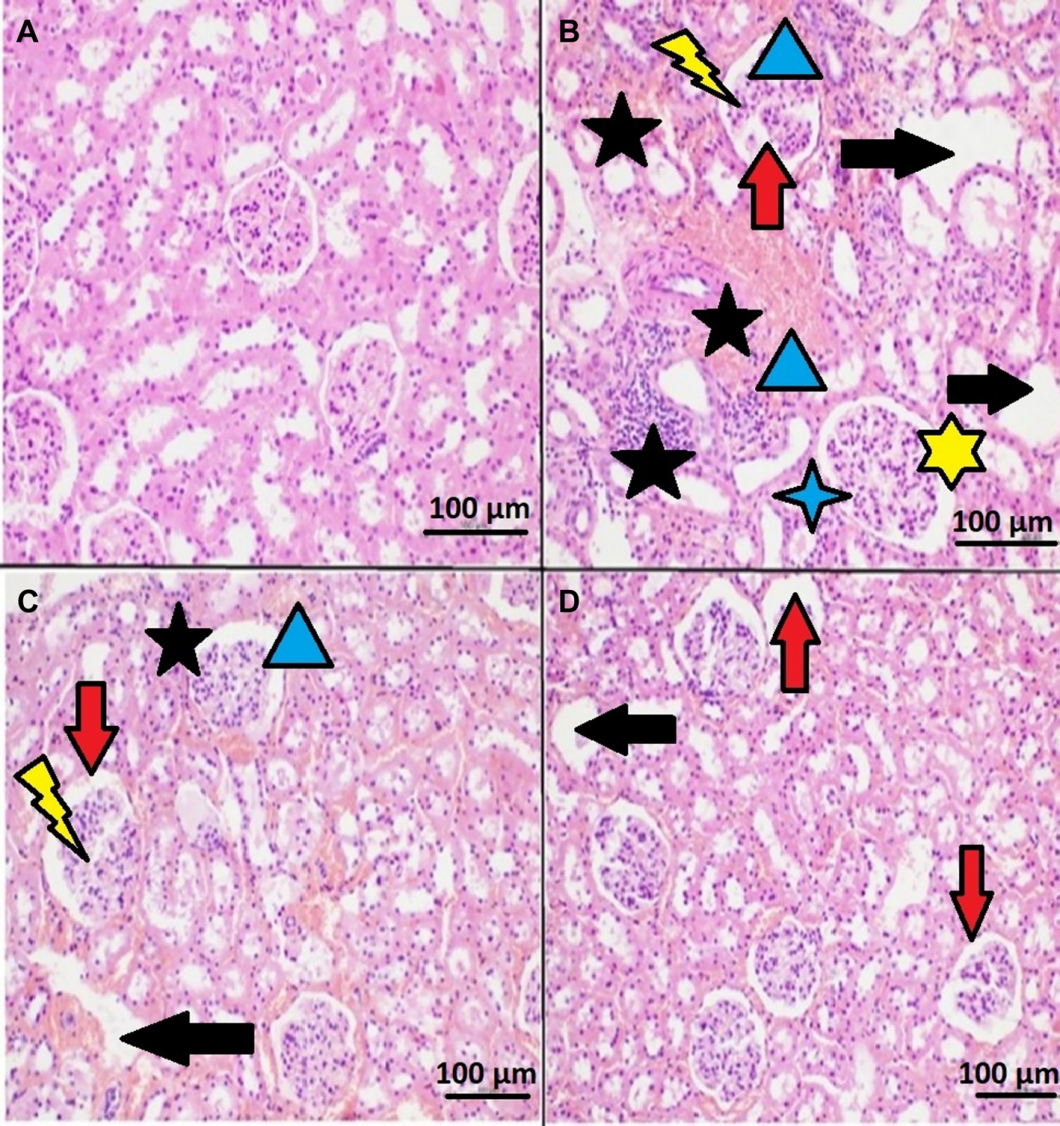
**Histopathological kidney tissue images.** (A) Control group; (B) IRI group; (C) 250 mg/kg arbutin+IRI (AR250+IRI) group; (D) 1000 mg/kg arbutin+IRI (AR1000+IRI) group. Horizontal black arrow: Dilation and necrosis; Vertical red arrow: Bowman’s capsule dilatation, flattening, and necrosis; Blue arrowhead-triangle: Degeneration and hemorrhage; 4-sided blue star: Sclerosis; 5-sided black star: Cellular infiltration, hemorrhage, and necrosis; 6-sided red star: Cortical-medullary congestion; Lightning-shaped yellow arrow: Enlargement in glomerular size. *Sections were taken at 2–3 µm thickness and stained with H&E. At least 10 high-power fields were examined for each kidney section. Photomicrographs of all kidney tissue samples were taken at ×200 magnification in H&E-stained preparations. Abbreviations: IRI: Ischemia–reperfusion injury; H&E: Hematoxylin and eosin.

Analyses employing H&E staining combined with light microscopy (H&E, ×200) revealed no histopathological abnormalities in the control group (Figure 4A). In contrast, the IRI group exhibited flattening, necrosis, degeneration, dilatation, glomerular necrosis and sclerosis, Bowman’s capsule dilatation, and interstitial hemorrhage (H&E, ×200) (Figure 4B). The AR250+IRI group displayed mild cortical-medullary congestion and a slight enlargement of glomerular size (H&E, ×200) (Figure 4C). The AR1000+IRI group demonstrated a similar histological appearance to that of the control group (H&E, ×200) (Figure 4D).

## Discussion

IRI is a multifaceted pathological process influenced by various factors, including metabolic stress, oxidative stress, inflammatory immune responses, leukocyte infiltration, and programmed cell death. IRI not only results from these factors but also induces a range of biological responses, such as oxidative stress, inflammation, and programmed cell death [28]. Among these responses, oxidative stress leads to the accumulation of reactive oxygen species (ROS) and the disruption of intracellular redox balance, compromising cell membrane integrity and initiating lipid peroxidation. Elevated lipid peroxidation results in structural alterations of cell membranes and mitochondrial dysfunction, ultimately culminating in oxidative cell death [29]. Malondialdehyde, a key biomarker of lipid peroxidation, significantly increases during IRI, and this elevation is closely linked to secondary organ damage [30].

In this study, malondialdehyde levels were elevated in the kidney tissue of rats subjected to IRI, while supplementation with varying doses of arbutin resulted in decreased malondialdehyde levels.

IMA, a biomarker for acute ischemia approved by the U.S. Food and Drug Administration, is instrumental in detecting acute ischemia prior to necrosis [31]. Furthermore, IMA levels rise in correlation with the duration of ischemia in IRI, a trend corroborated by histopathological evidence [32]. Another critical parameter that escalates due to inflammation and oxidative stress associated with IRI is myeloperoxidase, which contributes to the antimicrobial activity of neutrophils and is a vital component of the immune response in various inflammatory processes, including phagocytosis [33]. Myeloperoxidase interacts with the SOD substrate, superoxide, utilizing it as a cofactor to produce hypochlorous acid. In turn, SOD utilizes superoxide as a substrate to generate lower levels of ROS [34]. The overproduction of superoxide, which serves as both a substrate for SOD and a cofactor for myeloperoxidase, contributes to increased tissue damage in IRI [35].

In this study, myeloperoxidase levels were elevated in the kidney tissue of animals subjected to IRI, while levels decreased in animals that received varying doses of arbutin. Additionally, IMA and SOD (inhibition rate) levels increased in the kidney tissue of rats with IRI but decreased in those supplemented with 1000 mg/kg of arbutin.

A recent investigation indicated that arbutin, known for its anti-inflammatory and antioxidant properties, mitigates acute liver injury (ALI) by inhibiting apoptosis and inflammation [36]. Another study demonstrated that arbutin supplementation raised levels of GPx and catalase, thereby enhancing antioxidant activity [37]. Moreover, endogenous catalase is crucial for cellular antioxidant defenses and plays a significant role in preventing IRI [38]. In a study examining experimental testicular IRI, oxidative stress parameters increased with IRI, while arbutin supplementation elevated catalase levels and reduced oxidative stress markers [39]. Research on the protective effects of arbutin against ALI found that supplementation improved GPx and total antioxidant capacity levels, exhibiting hepatoprotective activity [40]. Furthermore, a study assessing the neuroprotective effects of arbutin against oxidative stress and neuroinflammation revealed that arbutin supplementation enhanced antioxidant status (e.g., glutathione, catalase, and SOD) and offered protection against stroke and ischemic injuries [41].

In this research investigating the potential nephroprotective effects of arbutin, catalase levels were diminished in the kidney tissue of rats with IRI, while they were significantly elevated in rats administered various doses of arbutin. Additionally, GPx levels were reduced in the kidney tissue of rats with IRI but increased in those supplemented with 1000 mg/kg of arbutin.

The initial phase of IRI is characterized by ischemia-mediated production of ROS in cells. This process activates pro-inflammatory signaling cascades, leading to the release of damage-associated molecular patterns, including TNF-α, interferon, interleukin-6, inducible nitric oxide synthase, and the TLR9/NF-κB pathway [42]. IRI induces morphological alterations, apoptosis, and inflammation in renal tissues. Notably, levels of pro-inflammatory cytokines such as IL-1β and TNF-α are significantly elevated in IRI conditions [43]. Numerous studies have reported increased levels of these pro-inflammatory cytokines in IRI, which is a prevalent cause of AKI [44–46].

In this study, IL-1β levels were found to increase in the kidney tissue of animals subjected to IRI, while these levels decreased in rats treated with various doses of arbutin. Additionally, TNF-α levels increased in the kidney tissue of IRI-affected animals and decreased in rats supplemented with 1000 mg/kg of arbutin.

The most widely accepted parameters for assessing renal failure include serum creatinine, urea, and glomerular filtration rate [47]. Increases in serum creatinine and urea levels indicate heightened susceptibility to tubular and tissue damage in IRI-induced AKI [48]. Furthermore, serum urea and creatinine levels are commonly used biochemical markers for evaluating renal function in experimental animal models subjected to IRI [49].

In the current study, serum creatinine levels rose in rats affected by IRI and decreased in those previously supplemented with varying doses of arbutin. Similarly, urea levels increased in the blood serum of IRI-affected animals but decreased in rats treated with 1000 mg/kg of arbutin.

An inverse relationship was observed between group scoring and levels of malondialdehyde, myeloperoxidase, IMA, TNF-α, IL-1β, SOD inhibition rate, creatinine, and urea. Conversely, a positive correlation was found between catalase and GPx levels.

Tubular cell death due to apoptosis and necrosis is a primary pathological characteristic of renal IRI [50]. AKI is typically marked by the rupture of plasma membranes in specific nephron segments, commonly referred to as “acute tubular necrosis” [51]. Histopathological findings associated with renal IRI include erythrocyte congestion, widespread cell swelling in both the medulla and cortex, significant cell apoptosis/necrosis in the medulla, and cast formation [52].

In this study, histopathological examination revealed flattening, necrosis, degeneration, dilation, glomerular necrosis and sclerosis, Bowman’s capsule dilation, and interstitial hemorrhage in the kidney tissue of IRI-injured animals. In contrast, no significant histopathological changes were observed in the kidney tissue of animals prophylactically treated with 1000 mg/kg of arbutin. Mild cortical-medullary congestion and slight glomerular enlargement were noted in the kidney tissue of rats treated with 250 mg/kg of arbutin.

This study has several limitations that warrant acknowledgment. First, the histological assessment was qualitative, and a blinded scoring system could not be implemented, limiting the quantitative interpretation of tissue damage. Second, only two widely spaced doses of arbutin were tested, which restricts the ability to establish a definitive dose–response relationship. Finally, the small sample size inherent to the preclinical animal model may limit the generalizability of the findings. Nevertheless, this study also possesses strengths, as its results can serve as a reference for future human clinical trials, quantitative histological assessments, or experimental studies exploring different dosage ranges. Therefore, this study significantly contributes to the direction of future research.

## Conclusion

Prophylactic supplementation with arbutin mitigated the effects of increased oxidative stress, impaired renal function, histopathological damage, and inflammation associated with IRI. Furthermore, arbutin supplementation was found to enhance the activities of antioxidant enzymes that were diminished due to IRI. These findings indicate that arbutin may possess protective effects against IRI, suggesting its potential as a supportive agent for prophylactic use.

## Data Availability

The datasets used and/or analyzed during the current study are available from the corresponding author on reasonable request.
